# Cytosolic protein delivery using pH-responsive, charge-reversible lipid nanoparticles

**DOI:** 10.1038/s41598-021-99180-5

**Published:** 2021-10-06

**Authors:** Yusuke Hirai, Hisaaki Hirose, Miki Imanishi, Tomohiro Asai, Shiroh Futaki

**Affiliations:** 1 Institute for Chemical Research, Kyoto University, Uji, Kyoto 611-0011 Japan; 2 Department of Medical Biochemistry, School of Pharmaceutical Sciences, University of Shizuoka, 52-1 Yada, Suruga-ku, Shizuoka, 422-8526 Japan

**Keywords:** Drug delivery, Drug delivery, Drug delivery

## Abstract

Although proteins have attractive features as biopharmaceuticals, the difficulty in delivering them into the cell interior limits their applicability. Lipid nanoparticles (LNPs) are a promising class of delivery vehicles. When designing a protein delivery system based on LNPs, the major challenges include: (i) formulation of LNPs with defined particle sizes and dispersity, (ii) efficient encapsulation of cargo proteins into LNPs, and (iii) effective cellular uptake and endosomal release into the cytosol. Dioleoylglycerophosphate-diethylenediamine (DOP-DEDA) is a pH-responsive, charge-reversible lipid. The aim of this study was to evaluate the applicability of DOP-DEDA-based LNPs for intracellular protein delivery. Considering the importance of electrostatic interactions in protein encapsulation into LNPs, a negatively charged green fluorescent protein (GFP) analog was successfully encapsulated into DOP-DEDA-based LNPs to yield diameters and polydispersity index of < 200 nm and < 0.2, respectively. Moreover, ~ 80% of the cargo proteins was encapsulated into the LNPs. Cytosolic distribution of fluorescent signals of the protein was observed for up to ~ 90% cells treated with the LNPs, indicating the facilitated endocytic uptake and endosomal escape of the cargo attained using the LNP system.

## Introduction

Proteins are among the essential molecules involved in maintaining cellular homeostasis^1^. Disorders in cellular protein expression levels or functions may lead to the impairment of cellular processes and the emergence of pathological conditions. Incorporation of bioactive proteins leads to the modulation of molecular production or molecular interplay in cells^2^. The development of effective in vivo protein delivery systems are thus in high demand^3^. Modification of proteins with peptides and polymers, which allow the delivery to specific organs/cells and the cell-permeation, is a practical approach^4,5^. However, appropriate methods of chemical modification, without compromising the structures and expected functions of the original proteins, are needed^6,7^. On the other hand, encapsulation of proteins into a suitable polymer- and lipid-based carrier is another promising approach, allowing delivery of intact proteins without needs of chemical modifications^8^.

Numerous polymer- and lipid-based carriers have been developed for the intracellular delivery of membrane-impermeable macromolecules such as nucleic acids, including small interfering RNA (siRNA), and antisense nucleic acids^9–13^. Lipid nanoparticles (LNPs) are one of the most studied carriers for nucleic acid delivery^14,15^. Strategies regarding their targeting to specific tissues or organs have also been exemplified. Three major issues have to be cleared in formulation of LNPs: (i) formulation of LNPs with defined particle sizes and dispersity, (ii) efficient encapsulation of cargo proteins into LNPs, and (iii) effective cellular uptake and endosomal release into the cytosol to obtain the expected activity. These are especially important when delivery of precious proteins including antibodies are intended. Considering the future applications of in vivo protein delivery in different aspects, the use of lipid nanoparticles (LNPs) as protein carriers is a practical choice^16,17^. LNPs are one of the most frequently studied carriers for gene delivery, strategies regarding their possible targeting to specific tissues or organs for gene delivery may also be employed^18,19^. However, considerably fewer approaches have been reported regarding intracellular protein delivery compared to nucleic acid delivery. Additionally, nanoparticles based on cationic lipids and polymers have been used for the delivery of proteins into cells^20–22^. Cationic carriers facilitate the intracellular delivery of proteins by effectively interacting with negatively charged cell surfaces, being taken up by endocytosis and eventually released into the cytosol. However, cationic carriers are generally associated with considerable cytotoxicity^20–22^. Carriers that have excellent cytosolic protein delivery efficacy, but are not cationic on the cell surface, are needed for clinical use.

**Figure 1:**
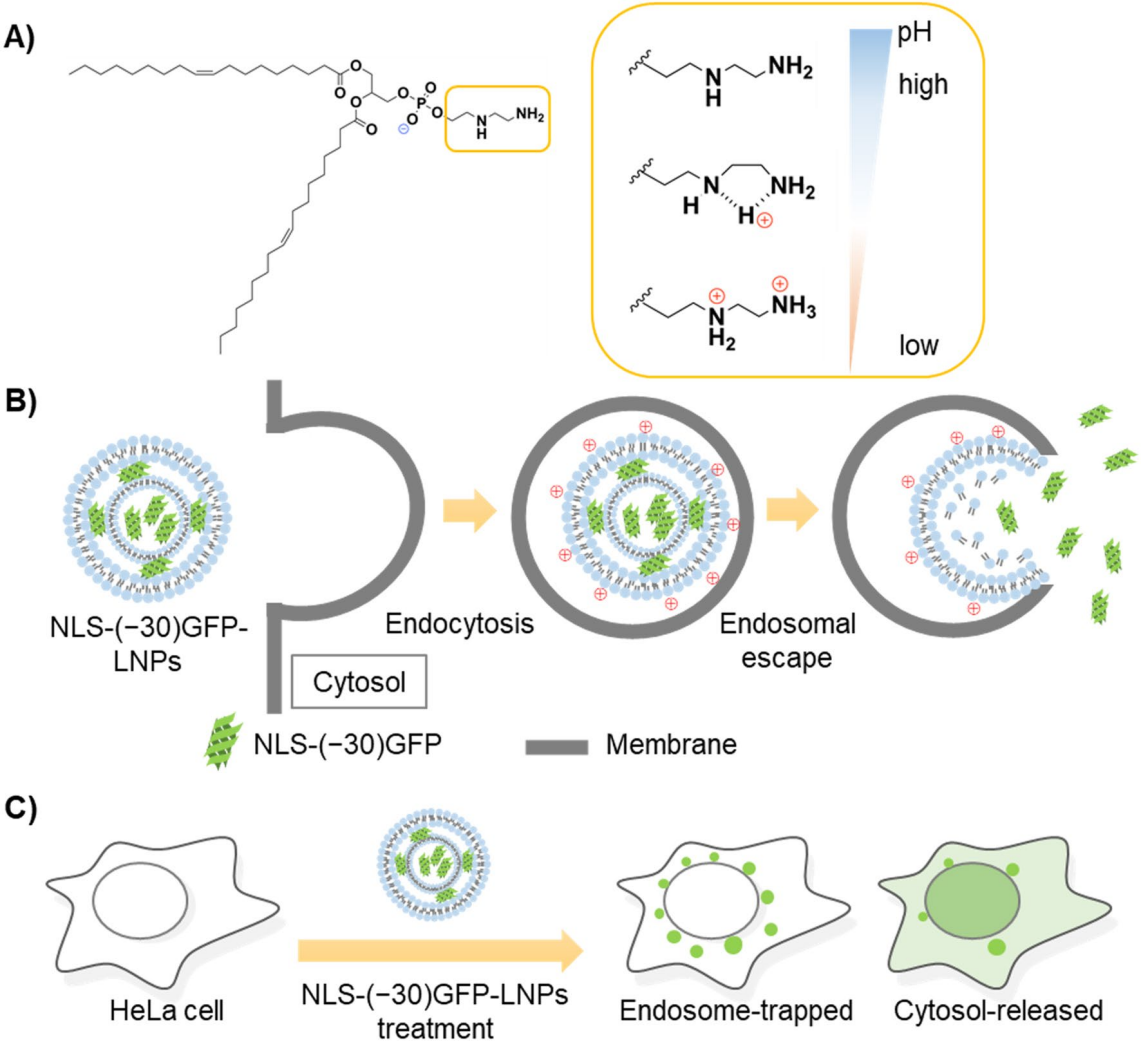
(**A**) Dioleoylglycerophosphate-diethylenediamine (DOP-DEDA) as a pH-sensitive, charge-reversible lipid and (**B**) hypothesized scheme of packaging and intracellular delivery of NLS-(− 30)GFP by DOP-DEDA-based carriers [NLS-(− 30)GFP-LNPs]. (**C**) Schematic illustration of the signals from endosome-trapped and cytosol-released NLS-(− 30)GFP.

We have developed a pH-responsive and charge-reversible cationic lipid nanoparticle (charge-reversible LNP) for siRNA delivery^23^. The salient feature of this LNP system is its marked encapsulation efficacy of siRNA (more than 95%) and efficient release to cytosol. This system employed a newly developed dioleoylglycerophosphate-diethylenediamine (DOP-DEDA) as the major component of the LNP (Fig. 1A). The lipid was designed to be negatively charged in the extracellular environment with neutral pH to avoid side effects arising from their interaction with cationic lipids (e.g., cytotoxicity, including the lung surfactant effect)^24,25^. However, once delivered into endosomes and exposed to an environment with reduced pH, the lipid becomes positively charged to interact with and rupture endosomal membranes (Fig. 1B). Effective siRNA delivery was achieved in human cancer cells using this LNP system, yielding a marked inhibition of cancer cell growth by inducing the knockdown of polo-like kinase-1^26^. Although this DOP-DEDA-based LNPs may have a potential applicability to protein delivery, no previous study has been made to evaluate this.

The aim of this study was to evaluate the ability of DOP-DEDA-based LNPs as a model carrier for intracellular protein delivery. The insights obtained in this study should also benefit the development of delivery systems based on other LNPs.

## Results and discussion

### Formulation of charge-reversible lipid-based nanoparticles encapsulating a negatively charged green fluorescent protein

Prior to evaluating the utility of LNPs for intracellular delivery, we first examined whether LNP-encapsulating proteins could be formulated using the DOP-DEDA system^23^. Considering that negatively charged siRNA is easily encapsulated into the DOP-DEDA system, the feasibility for encapsulating a negatively charged protein was first examined.

As a model protein bearing negative charges, the supernegative green fluorescent protein^27^ fused with a simian virus 40-derived nuclear localization signal segment (Pro-Lys-Lys-Lys-Arg-Lys-Val)^28^ [NLS-(− 30)GFP] was employed (protein net charge = –25). Punctate and dot-like signals should be obtained for the endosomally trapped fluorescently labeled proteins by confocal laser scanning microscopy (CLSM) (Fig. 1C). Due to the attachment with the NLS signal sequence, cytosol released protein can be accumulated into nucleus^29,30^. Therefore, percentage of cells bearing cytosol released protein can be evaluated.

**Figure 2:**
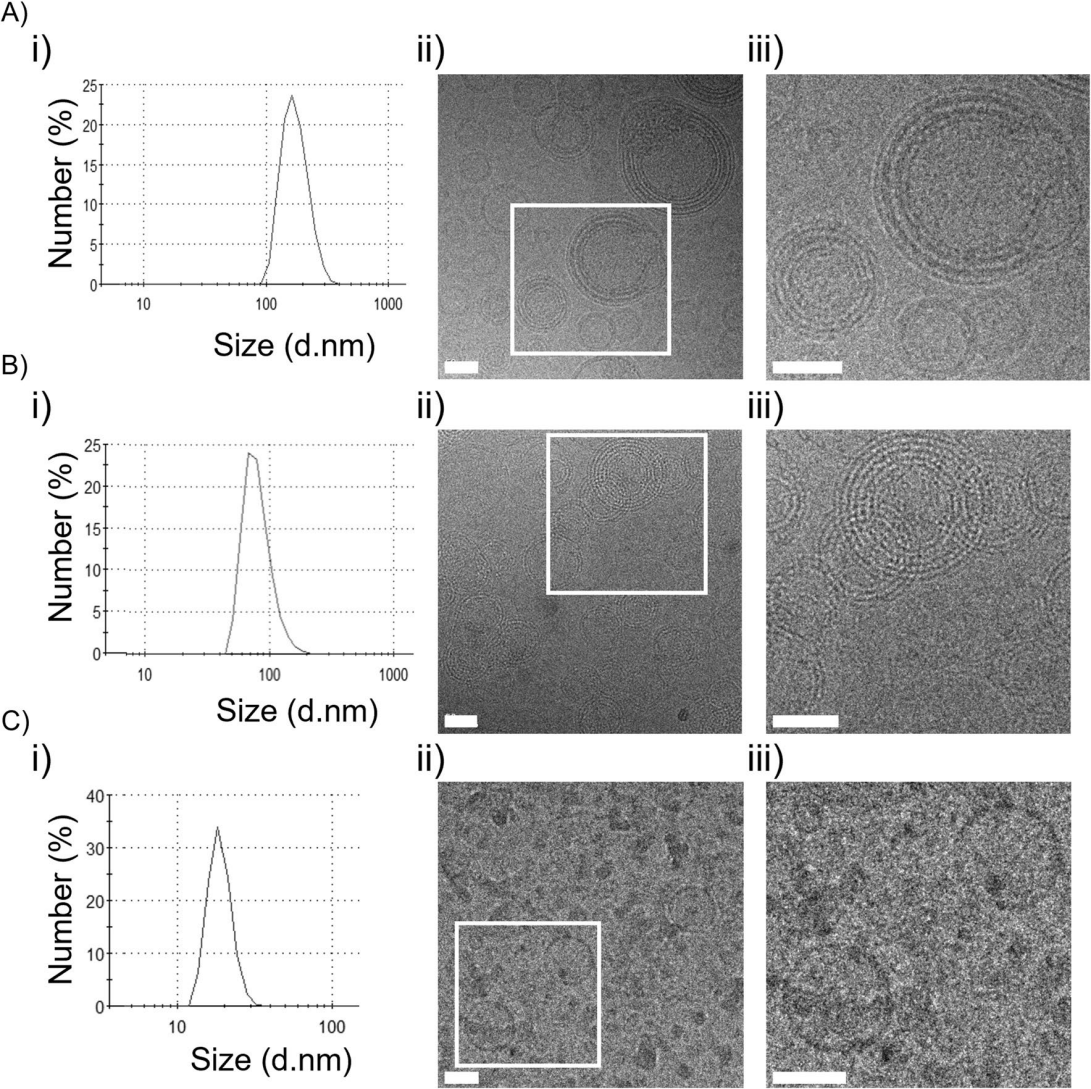
Size distribution and Cryo-TEM observation of NLS-(− 30)GFP-LNP formulations. (**A**) NLS-(− 30)GFP-LNPs (1:10), (**B**) NLS-(− 30)GFP-LNPs (1:50) and (**C**) LNPs formed without using NLS-(− 30)GFP [LNPs (no NLS-(− 30)GFP)]. (i) Size distribution of NLS-(− 30)GFP-LNPs analyzed using DLS. (ii) The morphology of NLS-(− 30)GFP-LNPs was observed via Cryo-TEM technology. (iii) The magnified image of (ii). Scale bars 50 nm.

Formulation of LNPs encapsulating NLS-(− 30)GFP [NLS-(− 30)GFP-LNPs] was conducted using the lipid composition used for siRNA delivery^23^, i.e., DOP-DEDA, dipalmitoylphosphatidylcholine (DPPC), cholesterol (Chol), and dimyristoylglycero-methoxypolyethyleneglycol molecular weight 5000 (DMG-PEG5k) at a 45/10/45/1 molar ratio. DMG-PEG5k was used to avoid the possible aggregation of LNPs^31–33^. To establish the NLS-(− 30)GFP-LNPs with preferable diameters and polydispersity index (PdI) (e.g., < 200 nm and < 0.2, respectively) from a drug delivery point of view^34^, those obtained from varying protein/lipid mass ratios (1:10 to 1:50) were first studied (Table 1) (see also SI for experimental details in LNP preparation). In all cases, LNPs with the desired diameters and PdIs were obtained. Analyzed via dynamic light scattering (DLS), LNPs with smaller diameters were obtained at lower NLS-(− 30)GFP concentrations. The diameter of the LNPs prepared from the mixture with a protein/lipid mass ratio of 1:10 was 172 ± 12 nm (Fig. 2A(i)), while those obtained from a 1:50 solution were 92 ± 11 nm (Fig. 2B(i)). The diameter of the LNPs without containing NLS-(− 30)GFP was 53 ± 5 nm (Fig. 2C(i)). Additionally, the PdIs for these LNPs were also in the preferable range of < 0.2. Considering the potential importance of protein/lipid ratios in membrane interaction, LNPs prepared from mixtures with protein/lipid mass ratios of 1:10 and 1:50 were employed in further studies, and their properties were analyzed [NLS-(− 30)GFP-LNPs (1:10) and NLS-(− 30)GFP-LNPs (1:50), respectively].

**Table 1:** Physicochemical characterization of NLS-(− 30)GFP-LNP by mixing NLS-(− 30)GFP with lipids at various mass ratios.

NLS-(− 30)GFP (μM)	Lipid (mM)	Mass ratio	Size (d.nm)	PdI
10.3	25	1:10	172 ± 12	0.156 ± 0.04
5.2	25	1:20	154 ± 32	0.134 ± 0.09
3.4	25	1:30	116 ± 20	0.164 ± 0.08
2.6	25	1:40	98 ± 8	0.154 ± 0.05
2.1	25	1:50	92 ± 11	0.126 ± 0.01

The lipid mixture was composed of DOP-DEDA, DPPC, and cholesterol at a 45:10:45 molar ratio, and 1 mol% DMG-PEG5k was added.

The mass ratios denote NLS-(− 30)GFP-LNP/total lipids (w/w). PdI = polydispersity index. Results are presented as the mean ± standard deviation (SD) of more than three independent experiments.

The morphologies of NLS-(− 30)GFP-LNPs (1:10) and NLS-(− 30)GFP-LNPs (1:50) were analyzed via Cryo-TEM (Fig. 2). The TEM images indicated that both LNPs had spherical multilamellar structures (Fig. 2A,B, (ii) and (iii)). LNPs prepared without NLS-(− 30)GFP [LNPs (no NLS-(− 30)GFP)] were also spherical but had a unilamellar structure (Fig. 2C, (ii) and (iii)). Their diameters were also smaller than that of the NLS-(− 30)GFP-LNPs (1:10). It was also reported by Cullis and his colleagues that LNP formulations comprised of siRNA and ionizable cationic lipid had multilamellar membrane structures where siRNA was sandwiched between closely apposed lipid monolayers, while LNPs without siRNA had unilamellar membrane structure^35^. These data suggest that the interaction of NLS-(− 30)GFP with DOP-DEDA may play a role in the formation of multiple lamellae and the entrapment of NLS-(− 30)GFP by the lipids within the particles.

### Effective intracellular delivery of NLS-(− 30)GFP attained by DOP-DEDA-based LNPs

**Figure 3:**
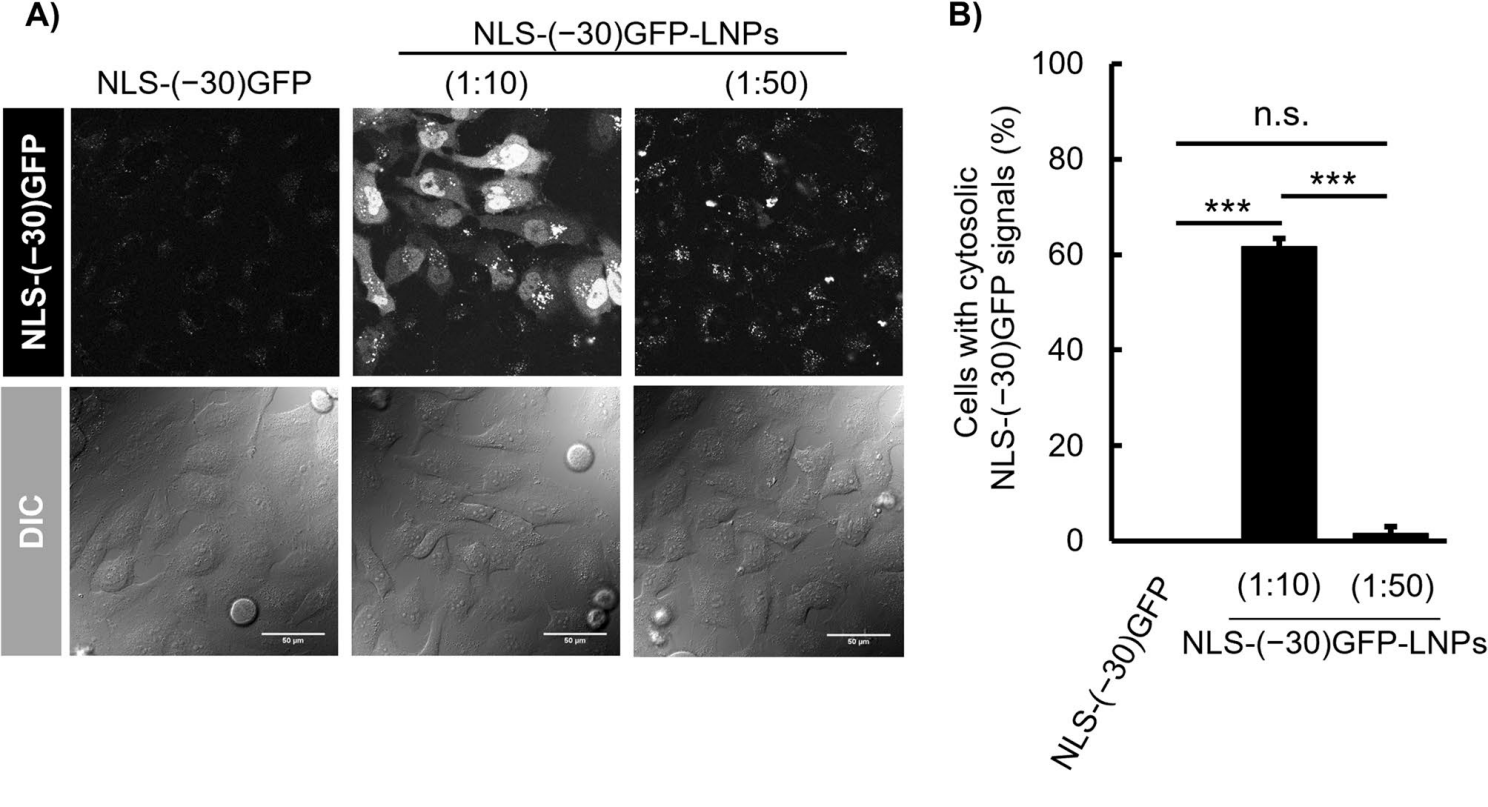
Cytosolic delivery of NLS-(− 30)GFP using DOP-DEDA-based LNPs. (**A**) Confocal laser scanning microscopy (CLSM) observation of the cytosolic appearance of NLS-(− 30)GFP after treatment with NLS-(− 30)GFP alone (left), NLS-(− 30)GFP-LNP (1:10) (center) and (1:50) (right) for 6 h. NLS-(− 30)GFP concentration = 2.5 μM. Scale bar, 50 μm. (**B**) Percentages of cells bearing cytosolic NLS-(− 30)GFP signals. Results are presented as mean ± standard deviation (SD) (n = 3). n.s., not significant; ***P < 0.001, (one-way analysis of variance [ANOVA] followed by Tukey–Kramer’s honestly-significant difference test).

The ability of DOP-DEDA-based LNPs for intracellular protein delivery was evaluated using CLSM. HeLa cells were treated with NLS-(− 30)GFP alone (i.e., without encapsulation into LNPs) or NLS-(− 30)GFP-LNPs with protein/lipid mass ratios of 1:10 and 1:50 in serum-containing medium for 6 h (Fig. 3). Cells treated with NLS-(− 30)GFP (2.5 µM) showed very little cytosolic or dot-like punctate signal in the cells, suggesting marginal cellular uptake of the NLS-(− 30)GFP protein (Fig. 3A, left). Concentration of NLS-(− 30)GFP-LNPs (1:10) and (1:50) was set to yield a final NLS-(− 30)GFP concentration of 2.5 µM in the medium (protein concentrations were calculated assuming that all proteins were encapsulated in the LNPs). Marked cytosolic and nuclear NLS-(− 30)GFP signals were observed in the cells treated with NLS-(− 30)GFP-LNPs (1:10) (Fig. 3A, center). Cytosolic release from endosomes is required prior to the translocation of NLS-(− 30)GFP to the nucleus (Fig. 1C). Therefore, nuclear NLS-(− 30)GFP signals are an indication of the cytosolic release of the protein (Figure S2). Approximately 60% of cells had NLS-(− 30)GFP signals in the nucleus following treatment with NLS-(− 30)GFP-LNPs (1:10) (Fig. 3B). Interestingly, NLS-(− 30)GFP-LNPs (1:50) yielded much less NLS-(− 30)GFP signals in the nucleus, but had punctate signals in the cells, suggesting the importance of the protein/lipid ratio to yield efficient cytosolic protein release (Fig. 3A, right and B). An increase in the DOP-DEDA ratio against NLS-(− 30)GFP does not necessarily lead to cytosolic release of the encapsulated proteins. It should be noted that in the case of nucleic acid delivery (e.g., siRNA and antisense oligonucleotides), the efficiency of endosomal escape is generally estimated to be less than a few percent^36^. Even under treatment conditions yielding the desired cell activity, CLSM analysis of fluorescently labeled nucleic acids often yield punctate cell distribution without spreading throughout the cell, indicating that the majorities of the nucleic acids are trapped in endosomes or form aggregate in cytosol^37–39^. The spread cytosolic signals of NLS-(− 30)GFP observed in this study thus support the suitability of the DOP-DEDA-based LNP system for intracellular protein delivery.

**Figure 4:**
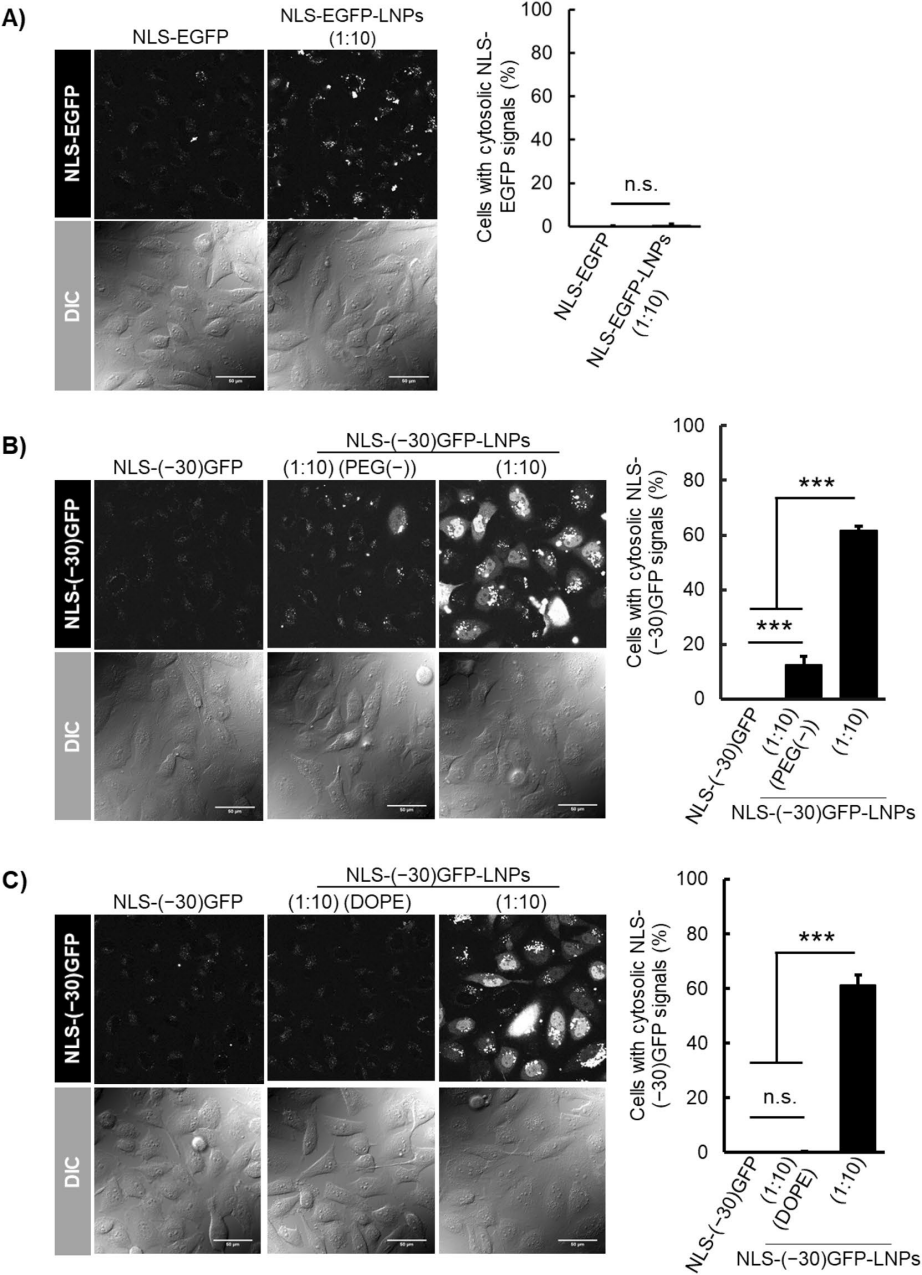
CLSM of cells treated with protein-encapsulating LNPs and the percentages of cells having cytosolic/nuclear localization of the proteins 6 h after treatment with the following: (**A**) NLS-EGFP alone (left) and NLS-EGFP-LNPs (1:10) (right); (**B**) NLS-(− 30)GFP alone (left), NLS-(− 30)GFP-LNPs (1:10) (PEG(–)) (center), and NLS-(–30)GFP-LNPs (right); (**C**) NLS-(− 30)GFP-LNPs (1:10) (DOPE) (center), and NLS-(–30)GFP-LNPs). The percentages of cells bearing cytosolic NLS-EGFP/NLS-(− 30)GFP signals are presented as the mean ± SD (n = 3). n.s., not significant; ***P < 0.001 (one-way analysis of variance (ANOVA) followed by Tukey–Kramer’s honestly-significant difference test). Protein concentration, 2.5 µM. Concentration of LNPs were set to yield 2.5 µM NLS-EGFP or NLS-(− 30)GFP when hypothesized that all the employed proteins for LNP preparation was encapsulated into the LNPs.

The use of cargos bearing highly negative charges is important for its efficient intracellular delivery using DOP-DEDA-based LNPs. This was suggested through the use of LNPs having the same lipid composition employed for NLS-(− 30)GFP-LNPs (1:10) but prepared by using enhanced green fluorescent protein^40^ (EGFP, pI = 5.6) tagged with NLS (i.e., NLS-EGFP-LNPs (1:10)). Although NLS-EGFP-LNPs (1:10) had preferable diameters (140 nm) and PdIs (0.153) (Table S3), no notable NLS-EGFP signals were observed in the cytosol and nucleus (Fig. 4A). Here NLS-EGFP-LNPs (1:10) was added to the cell culture medium to yield final protein concentration of 2.5 µM in the medium (protein concentration was calculated assuming that all proteins were encapsulated in the LNPs) as in the case of NLS-(− 30)GFP-LNPs. NLS-EGFP has a net charge of –9 and may less efficiently be encapsulated in DOP-DEDA-based LNPs than NLS-(− 30)GFP.

The surface of NLS-(− 30)GFP-LNPs (1:10) were decorated with PEG5k using DMG-PEG5k, which is important to define the particle structures of NLS-(− 30)GFP-LNPs (1:10) and increase the efficacy of the intracellular delivery^33^. NLS-(− 30)GFP-LNPs (1:10) were prepared without adding DMG-PEG5k to the lipid mixture (designated as NLS-(− 30)GFP-LNPs (1:10) (PEG(–))]. DLS analysis indicated that the diameter of NLS-(− 30)GFP-LNPs (1:10) (PEG(–)) reached > 2000 nm, suggesting aggregate formation (Table S3). A marginal level of cytosolic/nuclear localization in NLS-(− 30)GFP was also observed (Fig. 4B).

The importance of DOP-DEDA in the cytosolic/nuclear delivery of NLS-(− 30)GFP was also confirmed through the use of 1,2-dioleoyl-*sn*-glycero-3-phosphoethanolamine (DOPE)-based LNPs. DOPE is a lipid frequently employed for LNP formulations as a fusogenic lipid^31^. DOPE and DOP-DEDA share structural similarities through the ethanolamine and diethylenediamine moieties in their head groups, respectively. However, the amino group of DOPE is always positively charged under physiological conditions (p*K*a of ethanolamine, 9.5) and lacks pH sensitivity. LNPs with the same lipid composition other than the replacement of DOP-DEDA with DOPE [designated as NLS-(− 30)GFP-LNPs (1:10) (DOPE)] were similarly prepared; however, they had a diameter of 715 nm with a PdI 0.32, even though the LNPs contained DMG-PEG5k (1%) as a lipid component (Table S3). No significant NLS-(− 30)GFP signals were observed in the cells (Fig. 4C). We did not perform further studies to analyze the reasons for the poor NLS-(− 30)GFP delivery using the DOPE-based LNPs. However, the presence of weaker dot-like signals as seen in Fig. 4C, indicative of endosome-encapsulated NLS-(− 30)GFP, compared with the signals obtained after treatment with NLS-EGFP-LNPs (1:10) (Fig. 4A), may suggest a low endocytic efficacy due to the larger LNP size and lower encapsulation of NLS-(− 30)GFP into LNPs, suggestive of importance of pH-responsive charge-reversible characteristics of DOP-DEDA-based LNPs.

Overall, the above results suggested that NLS-(–30)GFP-LNPs (1:10) was most promising in cytosolic protein delivery. The encapsulation efficacy of cargo proteins in LNPs is one of the critical issues in this study. The encapsulation efficacy of NLS-(–30)GFP in NLS-(–30)GFP-LNPs (1:10) was as high as 78 ± 6% [mean ± standard deviation (SD)], n = 3) (see Supporting Information and Figure S1 for experimental details for the evaluation), further benefiting the use of DOP-DEDA-based LNP system for intracellular protein delivery.

### Time-course and concentration-dependence of cytosolic delivery using NLS-(− 30)GFP-LNPs (1:10)

**Figure 5:**
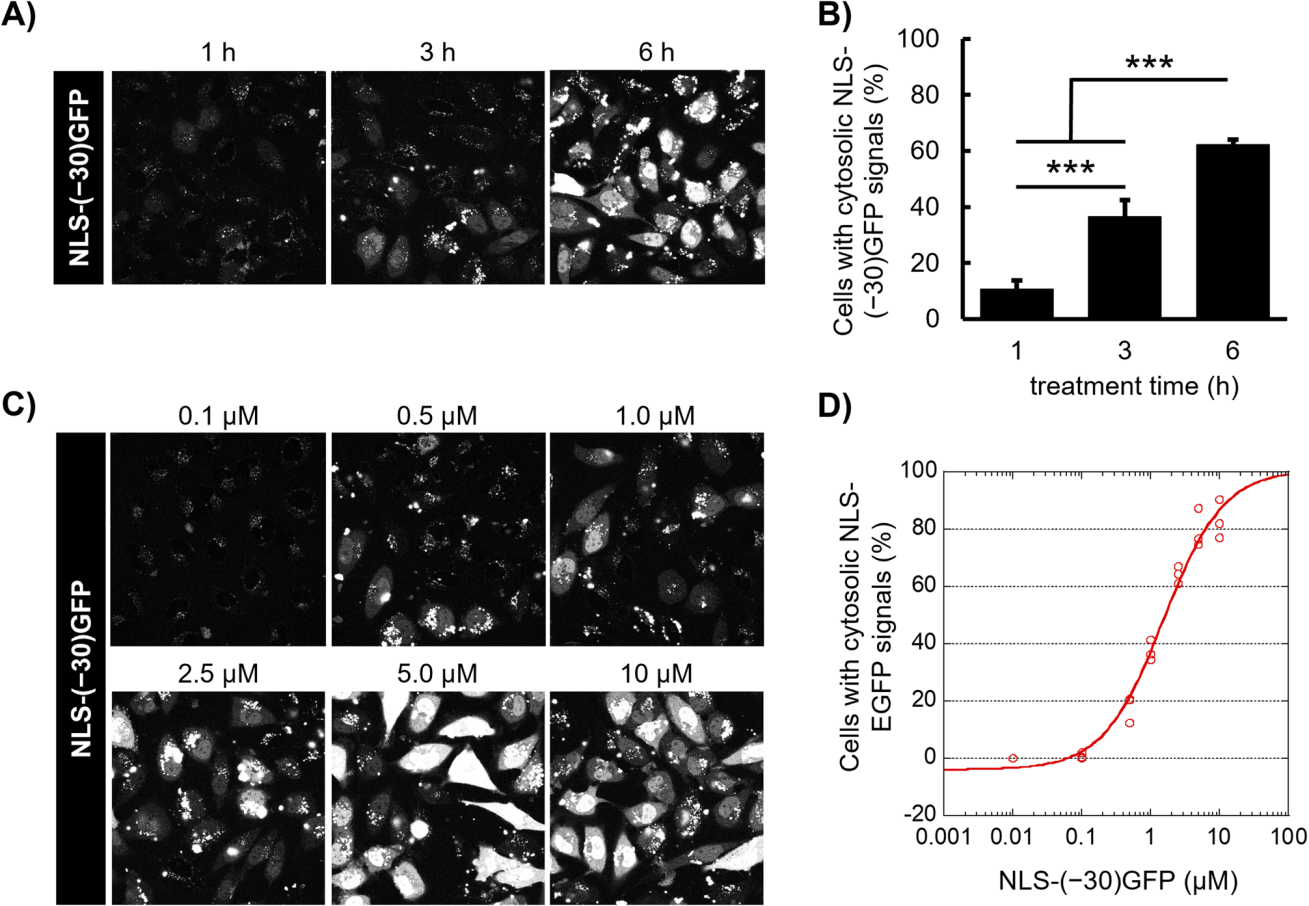
Time- and concentration-dependence of the cytosolic delivery of NLS-(− 30)GFP-LNPs. (**A**) CLSM observation of the cytosolic appearance of NLS-(− 30)GFP after treatment with NLS-(− 30)GFP-LNPs (1:10) (equivalent to 2.5 μM NLS-(− 30)GFP) for 1, 3, and 6 h. (**C**) CLSM observation of the cytosolic appearance of NLS-(− 30)GFP after treatment with NLS-(− 30)GFP-LNP (1:10) (NLS-(− 30)GFP concentration = 0.1, 0.5, 1.0, 2.5, 5.0 or 10 μM) for 6 h. (**B**, **D**) Percentages of cells bearing cytosolic NLS-(− 30)GFP signals. Results are presented as mean ± SD (n = 3). ***P < 0.001 (ANOVA followed by Tukey–Kramer’s honestly-significant difference test for (**B**)).

We next investigated the time-course and concentration dependence of the cytosolic delivery of NLS-(− 30)GFP using NLS-(− 30)GFP-LNPs (1:10) (Fig. 5A,B). HeLa cells were incubated with NLS-(− 30)GFP-LNPs for 1, 3, or 6 h at 37 °C, then the cells with NLS-(− 30)GFP signals were analyzed via CLSM. Punctate NLS-(− 30)GFP signals were predominantly observed in the cells 1 h after the addition of NLS-(− 30)GFP-LNPs (1:10). However, 3 h later, 40% of the cells exhibited cytosolic NLS-(− 30)GFP signals. There was a further increase in the percentage of NLS-(− 30)GFP-positive cells (60%) and signal intensity of NLS-(− 30)GFP at 6 h after incubation, suggesting the need for endosomal maturation to yield a low pH for the cytosolic release of NLS-(− 30)GFP from the DOP-DEDA-based LNPs.

There was a significant concentration dependency to yield a certain level of cytosolic delivery for NLS-(− 30)GFP (Fig. 5C,D). Although a marginal level of cytosolic delivery was attained after cellular treatment with NLS-(− 30)GFP-LNPs (1:10) (final protein concentration of 0.1 µM; protein concentration was calculated assuming that all proteins were encapsulated in the LNPs) for 6 h, ~ 90% of the cells showed signals of NLS-(− 30)GFP when treated to yield a final protein concentration of 10 µM (final lipid concentration, 4.8 mM). Additionally, NLS-(− 30)GFP-LNP treatment was not cytotoxic to HeLa cells in the above concentration range, as confirmed through the WST-8 assay, which is based on mitochondrial succinic dehydrogenase activity (Figure S3).

### Cellular uptake mechanisms of NLS-(− 30)GFP-LNP

The above time-course study suggested the involvement of endocytosis in the cytosolic delivery of NLS-(− 30)GFP using LNPs. This is further confirmed in the following section.

**Figure 6:**
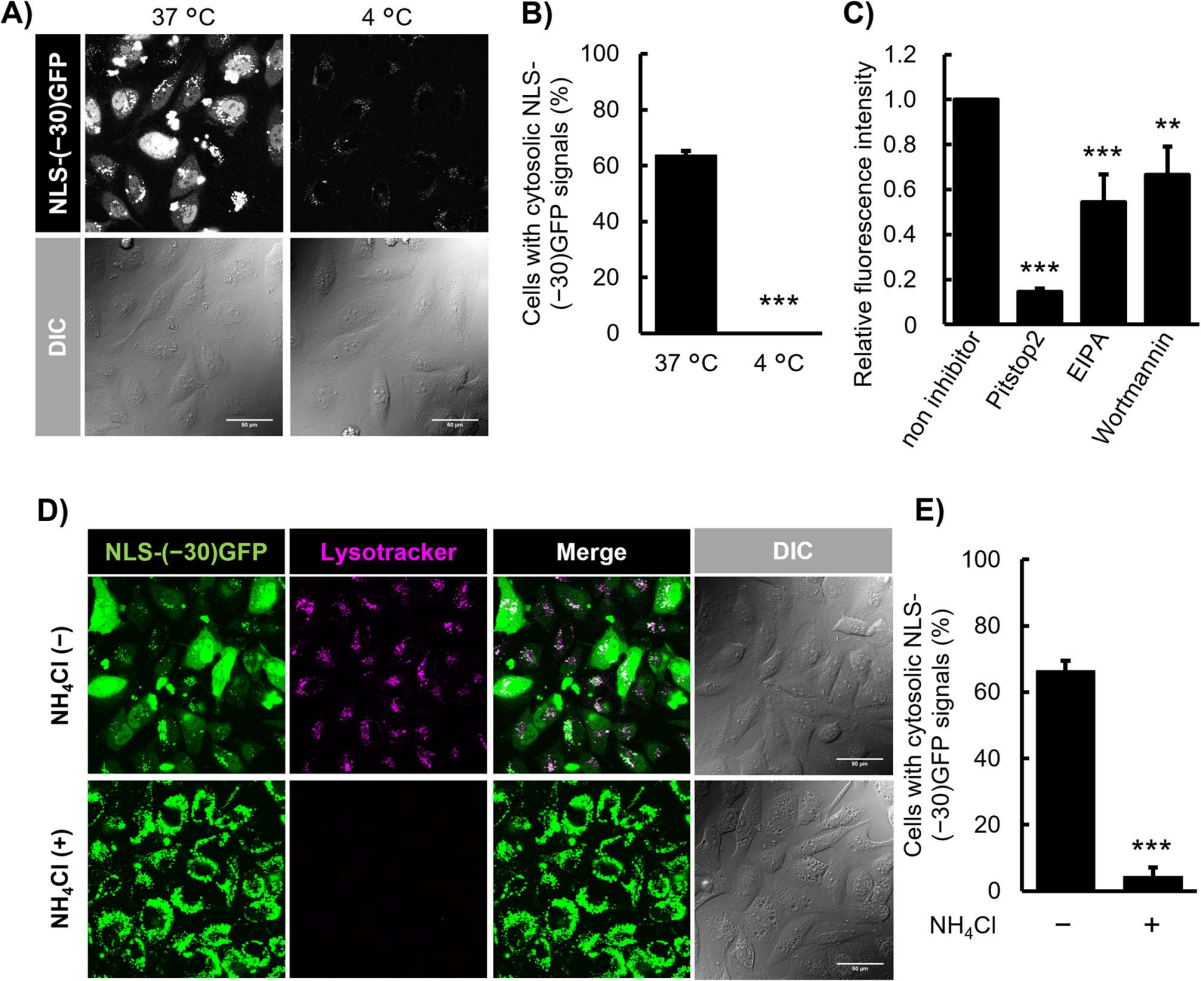
Endocytic uptake of NLS-(− 30)GFP-LNPs and the importance of endosome acidification in the cytosolic release of NLS-(− 30)GFP. (**A**) CLSM observation of the cytosolic appearance of NLS-(− 30)GFP after treatment with NLS-(− 30)GFP-LNPs (1:10) (equivalent to 2.5 μM NLS-(− 30)GFP) for 6 h at 37 °C or 4 °C. Scale bar, 50 μm. (**B**) Percentages of cells bearing cytosolic NLS-(− 30)GFP signals in (**A**). (**C**) Total cellular uptake of NLS-(− 30)GFP after treatment with endocytosis inhibitors: 30 μM of pitstop2 (a clathrin-mediated endocytosis inhibitor), 80 μM of EIPA (a macropinocytosis and clathrin-mediated endocytosis inhibitor) and 0.5 μM of wortmannin (a macropinocytosis related PI3K inhibitor). Cells were incubated with NLS-(− 30)GFP-LNP (1:10) (equivalent to 2.5 μM NLS-(− 30)GFP) for 6 h. (**D**) CLSM observation of the cytosolic appearance of NLS-(− 30)GFP after treatment with NLS-(− 30)GFP-LNP (1:10) (equivalent to 2.5 μM NLS-(− 30)GFP) for 6 h in the presence and absence of NH_4_Cl, an inhibitor of endosomal acidification. Scale bar, 50 μm. (**E**) Percentages of cells bearing cytosolic NLS-(− 30)GFP signals in (**D**). Results are presented as mean ± SD (n = 3). **P < 0.01; ***P < 0.001 (ANOVA followed by unpaired t-test for (**B**, **E**) and by Dunnett’s post hoc test (**C**) vs. non-inhibitor).

Endocytosis is an energy-driven cellular event that does not occur at 4 °C^41^. HeLa cells were treated with NLS-(− 30)GFP-LNP at 4 or 37 °C for 6 h. With a marked contrast compared to the treatment at 37 °C, no substantial NLS-(− 30)GFP signals, even those in dot-like, were observed after treatment at 4 °C (Fig. 6A,B). The effect of endocytosis inhibitors, such as pitstop2 (a clathrin-mediated endocytosis inhibitor)^42^, 5-(*N*-ethyl-*N*-isopropyl)amiloride (EIPA, an inhibitor of Na^+^/H^+^ exchanger and membrane ruffling)^43^ and wortmannin (a macropinocytosis inhibitor by blocking phosphatidylinocitol-3-kinase (PI3K))^44^, on the cellular uptake of NLS-(− 30)GFP-LNP was then analyzed (Fig. 6C). The cellular uptake of NLS-(− 30)GFP was evaluated using flow cytometry based on fluorescence intensity. HeLa cells were pre-treated with each endocytosis inhibitor (30 μM Pitstop2, 80 μM EIPA, or 0.5 μM wortmannin using dimethyl sulfoxide as a vehicle) for 30 min at 37 °C and then incubated with NLS-(− 30)GFP-LNP for 1 h at 37 °C in the presence of these inhibitors. After pitstop2 treatment, the cellular uptake of NLS-(− 30)GFP was 15% of that of untreated cells. Marked decreases in NLS-(− 30)GFP uptake were also observed in the presence of EIPA and wortmannin. These results suggest the possible involvement of clathrin-mediated endocytosis and macropinocytosis in the uptake of LNPs. The involvement of these endocytosis pathways has been suggested in the uptake of other LNPs. A detailed study on their similarities and differences would lead to the development of delivery systems with higher efficacy.

As expected from the time-course analysis of the cytosolic release of NLS-(− 30)GFP delivered by DOP-DEDA-based LNPs, endosomal maturation plays a crucial role. Because charge-reversible DOP-DEDA becomes protonated in acidic buffers, the surface of NLS-(− 30)GFP-LNPs should become positively charged along with endosomal maturation^23^. In contrast, the inner leaflet of endosomal membranes could also be negatively charged because of the abundance of negatively charged endosome-specific lipids, including bis(monoacylglycerol)phosphate (BMP)^45,46^.

To verify that endosomal acidification and the eventual protonation of DOP-DEDA are crucial for cytosolic NLS-(− 30)GFP release in the DOP-DEDA-based LNP system, endosomal acidification was blocked using ammonium chloride (NH_4_Cl)^47^ (Fig. 6D,E). HeLa cells were pre-treated with 25 mM NH_4_Cl for 30 min, then the cells were treated with NLS-(− 30)GFP-LNPs in the presence of 25 mM NH_4_Cl for 6 h. The prevention of endosomal acidification after NH_4_Cl treatment was confirmed by the loss of lysotracker signals, which are pH-sensitive dyes and indicators of acidic vesicular compartments (Fig. 6D, lysotracker, NH_4_Cl( +)).

In marked contrast to the cellular images of diffuse cytosolic/nuclear labeling in the absence of NH_4_Cl treatment (Fig. 6D, NLS-(− 30)GFP, NH_4_Cl( −)), NLS-(− 30)GFP signals were predominantly observed in the cytoplasmic area as large dot-like structures, and very little nuclear localization of NLS-(− 30)GFP was observed (Fig. 6D, NLS-(− 30)GFP, NH_4_Cl( +)). These data suggest that in NH_4_Cl-treated cells, the majority of NLS-(− 30)GFP remained trapped in endosomes without being liberated into the cytosol.

## Conclusions

There are numerous reports on LNP-based nucleic acid delivery. However, few reports have been published on the intracellular delivery of proteins, especially those including precise CLSM analyses of the cellular fates of cargo proteins.

In this study, we established an approach in producing LNPs based on the pH-sensitive, charge-reversible lipid DOP-DEDA, attaining efficient protein delivery into cells. The lipids employed for particle formation were composed of DOP-DEDA, DPPC, Chol, and DMG-PEG5k at a 45/10/45/1 molar ratio, where DOP-DEDA was found indispensable to attain efficient cytosolic release of the model cargo protein (NLS-(− 30)GFP). The obtained LNPs had diameters and PdIs in the preferable range for drug delivery (< 200 nm and < 0.2, respectively). DMG-PEG5k played an important role in obtaining these PdIs. Although negative charges were needed in the cargo protein for effective encapsulation into the LNPs, almost 80% of the NLS-(− 30)GFP used in the formulation was incorporated into LNPs. When administered to the cells, successful delivery of NLS-(− 30)GFP-LNPs to the cell interior was observed in ~ 90% of the cells evaluated by CLSM analysis. We do not presume that all NLS-(− 30)GFP has a proper folding structure after being released from LNP into the cytosol. However, the CLSM analysis showed that a significant amount of NLS-(− 30)GFP maintained its active structure, which is important from the perspective of drug delivery. The high ratio of the cargo proteins charged into the LNPs and released into the cytosol suggest the promise of this DOP-DEDA-based LNP system as a vehicle for the intracellular delivery of bioactive proteins.

The possible involvement of endocytosis, including clathrin-mediated endocytosis and macropinocytosis, were suggested as the mechanisms of the cellular uptake of NLS-(− 30)GFP-LNPs. Endosomal acidification also plays a role in the cytosolic release of NLS-(− 30)GFP from endosomes, as was suggested in a time-course study of the cellular distribution of NLS-(− 30)GFP and the prevention of endosome acidification under NH_4_Cl treatment. These studies on uptake mechanisms suggest the validity of our design concept.

One of the future goals of this research is the intracellular delivery of antibodies, including low molecular weight antibodies (such as single chain variable fragments (scFv) and nanobodies). Currently, antibody therapeutics used in clinical practice are limited to targeting molecules outside the cell. If this LNP system could deliver antibodies into the cytosol, it could extend the scope of antibody therapy to molecules inside the cell, potentially leading to the treatment of unmet medical needs, including cancer. It has been reported that nanocarriers bearing diameters of 50–100 nm generally exhibit long blood half-life and preferentially accumulate at solid tumors, without efficiently excreted in the urine or phagocytosed by macrophages^48,49^. The diameter of the LNPs estimated in this study (100–170 nm) was slightly larger than these, but still within the acceptable range, suggesting the potential in vivo applicability of this approach.

This time NLS-(− 30)GFP was employed as a model cargo. The hydrodynamic radius of the green fluorescent protein is reported to be about 2.3 nm^50^, which is consistent with the distance between adjacent lipid monolayers of LNPs estimated from TEM images (about 5 nm) (Fig. 2A-(iii)), suggesting that NLS-(− 30)GFP may act as an adhesive and form multilayered structures. This also suggests that LNPs may be able to encapsulate larger sized proteins in larger spaced multilayer structures, by further optimizing formulation methods including lipid compositions if necessary. In this study, proteins with negative charges are used for encapsulation into LNPs. Further work is needed to encapsulate cargo proteins that do not have a negative charge. The use of tag sequences with negative charges may be one possible approach.

As mentioned above, there are still challenges to be overcome. Nevertheless, we believe that this study is an important first step towards understanding intracellular protein delivery using LNPs.

## Methods

### Materials

pET-6 × His-(− 30)GFP was a gift from David Liu (Addgene plasmid # 62,936; http://n2t.net/addgene:62936; RRID:Addgene_62936) ^19^. Primers were purchased from Eurofins Genomics (Tokyo, Japan). The charge-reversible lipid DOP-DEDA, (dioleoylglycerophosphate-diethylenediamine conjugate) was a kind gift from Nippon Fine Chemical Co. (Osaka, Japan). Dipalmitoylphosphatidylcholine (DPPC) and 1, 2-Dimyrustoyl-*rac*-glycero-3-methylpolyoxyethylene-polyethyleneglycerol chain, molecular weight 5000 (DMG-PEG5k) were purchased from NOF Corporation (Tokyo, Japan). Cholesterol was purchased from Sigma-Aldrich (St. Louis, MO, USA).

### Construction of a plasmid encoding NLS-(− 30)GFP and its protein expression and purification

To construct a plasmid for the recombinant expression of the nuclear localization signal (SV40NLS; sequence, PKKKRKV)-fused super-negatively charged GFP [pET-*6* × *His-SV40NLS-(− 30)GFP*], the 6 × His-SV40NLS coding sequence was obtained by annealing the following oligonucleotides after phosphorylation at the 5′-ends with T4 PNK (New England Biolabs, Ipswich, MA, USA): 5′- CATGGGTCATCACCACCACCATCACGGTGGCCCTAAGAAGAAACGTAAGGTCGGAGGCAGCC-3′; 5′- CTAGCGCTGCCTCCGACCTTACGTTTCTTCTTAGGGCCACCGTGATGGTGGTGGTGATGACC-3′. This sequence was inserted between the *Nco*I and *Nhe*I restriction enzyme sites of pET-6 × His-(− 30)GFP.

*E. coli* BL21 (DE3)-competent cells (Nippon Gene, Tokyo, Japan) were transformed with pET-6 × His-SV40NLS-(− 30)GFP. The resulting expression strain was inoculated into 1 L Luria–Bertani broth (Thermo Fisher Scientific, Waltham, MA, USA) containing 100 mg/mL ampicillin until it reached an OD_600_ of 0.6. Protein expression was induced by adding 0.5 mM isopropyl-β-d(–)-thiogalactopyranoside (Fujifilm Wako Pure Chemical Corporation, Osaka, Japan) and subsequent incubation at 18 °C and 100 rpm for 18 h. Bacterial cells were collected via centrifugation using a JLA-9.100 rotor (Beckman Coulter, Brea, CA, USA) at 4,000 rpm for 10 min. The pellet was processed immediately or stored at -80 °C until further use.

To purify the NLS-(− 30)GFP obtained, the pellets were resuspended in lysis buffer (20 mM Tris–HCl, 300 mM NaCl, 10 mM imidazole, pH 7.5). The cells were lysed via sonication (2-min on and 2-min off cycle total at 5 times output, on ice), the soluble lysates were obtained via centrifugation at 20,000 × *g* for 30 min at 4 °C, and the supernatant was filtered through a 0.45 μm syringe filter. The cell lysate was incubated with 2 mL nickel-nitriloacetic acid (Ni–NTA) agarose (Qiagen, Hilden, Germany) at 4 °C for 1 h to capture NLS-(− 30)GFP with a 6 × His-tag. The resins were washed twice with wash buffer (20 mM Tris–HCl, 300 mM NaCl, 20 mM imidazole, pH 7.5). NLS-(− 30)GFP was eluted with an elution buffer (20 mM Tris–HCl, 300 mM NaCl, 250 mM imidazole, pH 7.5) and concentrated using an Amicon centrifugal filter 10 MWCO (Merck Millipore, Burlington, MA, USA) with phosphate-buffered saline without containing Ca^2+^ and Mg^2+^ (PBS(–)). The eluate was further purified using a HiTrap Q HP anion exchange column (GE Healthcare Bioscience, Piscataway, NJ, USA). NLS-(− 30)GFP was eluted with a purification buffer (50 mM Tris–HCl, 0.1, or 1 mM NaCl, 1 mM dithiothreitol (DTT), pH 8.0) containing a linear NaCl gradient from 0.1 to 1 M over five column volumes. The eluted fractions containing NLS-(− 30)GFP were buffer exchanged with PBS(–) and concentrated to 1.0 mg/mL as quantified using a bicinchoninic acid (BCA) assay (Thermo Fisher Scientific).

### Preparation of lipid nanoparticle (LNP)-encapsulated NLS-(− 30)GFP

DOP-DEDA, DPPC, cholesterol, and DMG-PEG5k were dissolved in chloroform (Fujifilm Wako Pure Chemical Corporation) and stored at − 30 °C. These lipids were mixed in a flask at a molar ratio of DOP-DEDA/DPPC/Cholesterol/DMG-PEG5k = 45/10/45/1 and an appropriate volume of *t*-butanol was added (Fujifilm Wako Pure Chemical Corporation). Chloroform was then removed using a rotary evaporator and the resulting solution was lyophilized. The lipid product was dissolved in *t*-butanol to a final lipid concentration of 25 mM. NLS-(− 30)GFP was dissolved in 1 mM citrate buffer (pH 5.0). The lipids and NLS-(− 30)GFP solutions were separately heated to 40 °C and then mildly mixed by pipetting for 30 times. The mixture was then dialyzed against ultrapure water (more than 1,000 times volume, molecular weight cut-off of 12–14 kDa; Spectrum Laboratories Inc., Rancho Dominguez, CA) to remove the *t*-butanol. The particle size and polydispersity index (PdI) were measured via dynamic light scattering using a Zetasizer Nano S (Malvern, Worcestershire, UK).

To study the effect of the pH of the citrate buffer on the NLS-(− 30)GFP-LNP formulation, LNPs were prepared using 1 mM citrate at pH 4.5—6.0 similarly as described above (Table S1).

To study the Effect of volume ratios of the aqueous/organic phases on the NLS-(− 30)GFP-LNP formulation, 3 to 10 volumes of aqueous phase (1 mM citrate buffer, pH 5.0) were added to a fixed volume of *t*-butanol (lipid concentration, 25 mM). The amount of total protein was also fixed to retain a protein/lipid mass ratio of 1:10 (Table S2).

For the characterization of NLS-(− 30)GFP-LNP formulated from NLS-(− 30)GFP and lipids at various mass ratios, NLS-(− 30)GFP in 1 mM citrate buffer (pH 5.0) was mixed with the lipid mixture of DOP-DEDA/DPPC/Chol/DMG-PEG5k (45/10/45/1 molar ratio) in *t*-butanol. The volumes of the aqueous and organic phases were fixed at 5:1. Starting from NLS-(− 30)GFP at a concentration of 2.1 μM (protein/lipid mass ratio of 1:50), increasing concentrations (up to 10.3 μM, protein/lipid mass ratio of 1:10) of NLS-(− 30)GFP solution were employed. LNPs with the desired diameters and PdIs were then obtained from these mixtures.

### Calculation of NLS-(− 30)GFP encapsulation efficiency in LNP formulations

The NLS-(− 30)GFP encapsulation efficiencies in the NLS-(− 30)GFP-LNP formulations were determined as follows. NLS-(− 30)GFP-LNP formulations were prepared, then, half of the respective LNP samples were adjusted to include 2% SDS and stored at 4 °C, and the other halves were precipitated via ultracentrifugation (100,000 × *g*, 4 °C, 2 h). The supernatants were removed, and the pellets were dissolved with 2% SDS to match the volume of the non-ultracentrifuged LNP samples. The same volume of LNP samples was applied onto a 10% acrylamide gel and resolved using sodium dodecyl sulfate–polyacrylamide gel electrophoresis (SDS-PAGE). Lastly, to detect the NLS-(− 30)GFP encapsulated in LNP formulations, Coomassie Brilliant Blue staining was performed. The encapsulation efficiency was calculated using Eq. (1), where P_pellet_ is the amount of NLS-(− 30)GFP in the pellet after ultracentrifugation and P_total_ is the amount of NLS-(− 30)GFP in the non-ultracentrifuged LNP samples.

1 $$\% \;{\text{of}}\;{\text{encapsulation}}\;{\text{efficiency}}\;{\text{of}}\;{\text{NLS}} - \left( { - {3}0} \right){\text{GFP}}\;{\text{in}}\;{\text{LNP}}\;{\text{formulation}} = {\text{P}}_{{{\text{pellet}}}} /{\text{P}}_{{{\text{total}}}} \times {1}00$$

### Cryogenic transmission electron microscopy (Cryo-TEM)

To observe the morphology of NLS-(− 30)GFP-LNPs, Cryo-TEM images were collected using a JEOL/JEM-2100F(G5). The LNP suspension was concentrated to a final concentration of 20 mg/mL of total lipids. After a small amount (3 – 5 μL) of the LNP suspension was placed on a TEM copper grid covered by a porous carbon film, the excess solution on the grid was immediately plunged into liquid propane in a cryofixation apparatus (Reichert KF-80, Leica Microsystems, Wetzlar, Germany) to generate vitreous ice. Then, the ice was transferred onto the specimen stage of a Cryo-TEM operated at an acceleration voltage of 200 kV, and Cryo-TEM images were obtained at liquid helium temperature (4.2 K).

### Cell culture

HeLa cells (human epithelial carcinoma cell line) obtained from the European Collection of Authenticated Cell Cultures (ECACC, Salisbury, UK, no. 93021013) were cultured in α-minimum essential medium (α-MEM) supplemented with 10% (v/v) heat-inactivated bovine serum (BS) (α-MEM( +)). HeLa cells were maintained at 37 °C in a humidified 5% CO_2_ incubator, and subculture was conducted every 2–4 days.

### Microscopic observation of cellular uptake and cellular distribution of NLS-(− 30)GFP delivered by LNP formulation

HeLa cells were seeded onto 35 mm glass-bottom dishes (Iwaki, Tokyo, Japan) and cultured at 37 °C with 5% CO_2_ for 24 h up to about 60% confluence. Cells were washed twice with PBS(–) and were then incubated with LNPs encapsulating 0—10 μM NLS-(− 30)GFP in α-MEM( +) for 6 h. Then, the cells were washed with PBS(–) containing 0.5 mg/mL heparin and incubated at 37 °C in α-MEM( +) for 12 h. After washing twice with PBS(–), the nuclei were stained with 5 μg/mL Hoechst 33,342 (Thermo Fisher Scientific), and α-MEM( +) was added to the dishes. The cellular localization of NLS-(− 30)GFP was then analyzed via live-cell imaging using FV1000 or FV3000 confocal laser scanning microscopy (CLSM) (Olympus, Tokyo, Japan). The number of cells with NLS-(− 30)GFP signals detected in the nuclei, which was defined as successful cytosolic delivery (referred to as cells with cytosolic NLS-(− 30)GFP in this study), and > 400 cells were counted for each sample.

For the time-course analysis (Fig. 5A), HeLa cells were treated with NLS-(− 30)GFP-LNP at a concentration of 2.5 μM as NLS-(− 30)GFP in α-MEM( +) for 1, 3, or 6 h at 37 °C. The cells were then washed with PBS(–) containing 0.5 mg/mL heparin and incubated at 37 °C in α-MEM( +) for 12 h and observed with CLSM.

To evaluate whether the cellular uptake of NLS-(− 30)GFP-LNP relies on energy-dependent pathways, HeLa cells were pre-treated with α-MEM( +) for 0.5 h at 4 °C before the addition of NLS-(− 30)GFP-LNP. Next, LNPs encapsulating 2.5 μM NLS-(− 30)GFP in α-MEM( +) was added to HeLa cells and then incubated for 6 h at 4 °C. The cells were then washed with PBS(–) containing 0.5 mg/mL heparin and incubated at 4 °C in α-MEM( +) for 12 h and observed via CLSM.

To evaluate the intracellular delivery of NLS-(− 30)GFP via NLS-(− 30)GFP-LNP under the presence of an endosomal acidification inhibitor, HeLa cells were pretreated with or without 25 mM of ammonium chloride (NH_4_Cl) in α-MEM( +) at 37 °C for 0.5 h, NLS-(− 30)GFP-LNP was then added at concentration of 2.5 μM as NLS-(− 30)GFP in 25 mM NH_4_Cl and incubated for 6 h at 37 °C. The cells were then washed with PBS(–) containing 0.5 mg/mL heparin and incubated at 37 °C in α-MEM( +) for 12 h with or without 25 mM of NH_4_Cl and then observed using CLSM.

### WST-8 assay

Cell viability was determined using the Cell Counting Kit-8 (CCK-8) (Dojindo Lab., Kumamoto, Japan), following the manufacturer’s protocol. Briefly, HeLa cells were treated with NLS-(− 30)GFP-LNPs at concentrations of 0, 2.5, 5.0, 10, or 20 μM NLS-(− 30)GFP for 6 h in α-MEM( +). Cells were washed twice with PBS(–) containing 0.5 mg/mL heparin, WST-8 assay reagent was added, and the cells were incubated for another 1 h. The absorbance at 450 nm was measured.

### Flow cytometry analysis of cellular uptake of NLS-(− 30)GFP delivered by LNPs with endocytosis inhibitors

HeLa cells (8 × 10^4^ cells/well) were seeded onto 24-well plates (Iwaki) and cultured at 37 °C with 5% CO_2_ for 24 h. Cells were washed twice with PBS(–). To evaluate the cellular internalization pathway of NLS-(− 30)GFP-LNP, HeLa cells were pre-treated with endocytosis inhibitors, 30 μM of Pitstop2 (a clathrin-mediated endocytosis inhibitor), 80 μM of 5-(*N*-ethyl-*N*-isopropyl)amiloride (EIPA, a macropinocytosis inhibitor), or 500 nM of wortmannin (a macropinocytosis-related PI3K inhibitor) in α-MEM(–) for 0.5 h at 37 °C. Next, NLS-(− 30)GFP-LNPs (equivalent to 2.5 μM NLS-(− 30)GFP) in α-MEM( +) were added to HeLa cells and incubated with the endocytosis inhibitors for 1 h at 37 °C. The cells were then washed twice with PBS(–) containing 0.5 mg/mL heparin and incubated with 0.01% trypsin for 10 min at 37 °C. Suspended cells were collected in 1.5 mL tubes, washed twice with PBS(–), and subjected to flow cytometry analysis using an Attune NxT flow cytometer (Thermo Fisher Scientific). The analysis was performed on 10,000 gated events per sample.

## Supplementary Information

Supplementary Information.
