# Supplementary material for: Cytosolic protein delivery using pH-responsive, charge-reversible lipid nanoparticles
Source: Sci Rep. 2021 Oct 6;11:19896. doi: 10.1038/s41598-021-99180-5 (PMC8494842; doi:10.1038/s41598-021-99180-5)
Supplement: Supplementary file 1 — Supplementary Information. [file 41598_2021_99180_MOESM1_ESM.pdf]

Cytosolic protein delivery using pH-responsive, charge-reversible lipid nanoparticles

Yusuke Hirai,[a] Hisaaki Hirose,[a] Miki Imanishi,[a] Tomohiro Asai,[b]* Shiroh Futaki[a,]*

[a] *Institute for Chemical Research, Kyoto University, Uji, Kyoto 611-0011, Japan*

[b] *Department of Medical Biochemistry, University of Shizuoka School of Pharmaceutical Sciences, 52-1 Yada, Suruga-ku, Shizuoka 422-8526, Japan*

Supplementary Information

Figures S1-S3

Tables S1-S3

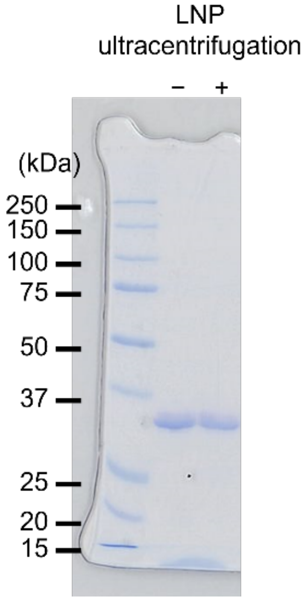

Figure S1. Evaluation of NLS-(−30)GFP encapsulation efficacy using the NLS-(−30)GFP-LNPs (1:10) formulation.

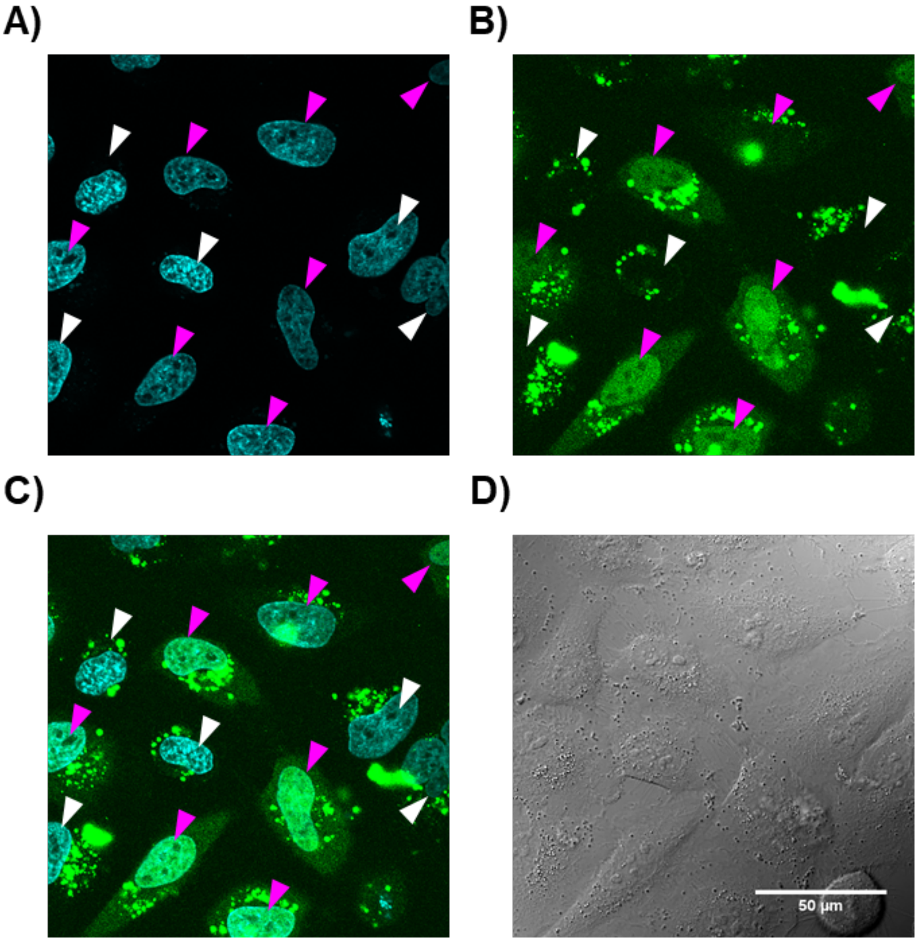

Figure S2. CLSM analysis of the cytosolic delivery of NLS-(−30)GFP using NLS-(−30)GFP-LNPs (1:10). (A) Nuclear staining with Hoechst 33342. (B) NLS-(−30)GFP signals. (C) Merged image of (A) and (B). (D) Differential interference contrast (DIC) image. Scale bar, 50 µm. The magenta arrowheads indicate cells with NLS-(−30)GFP delivered into the nuclei. Arrowheads in white indicate cells without localization of NLS-(−30)GFP in the nuclei.

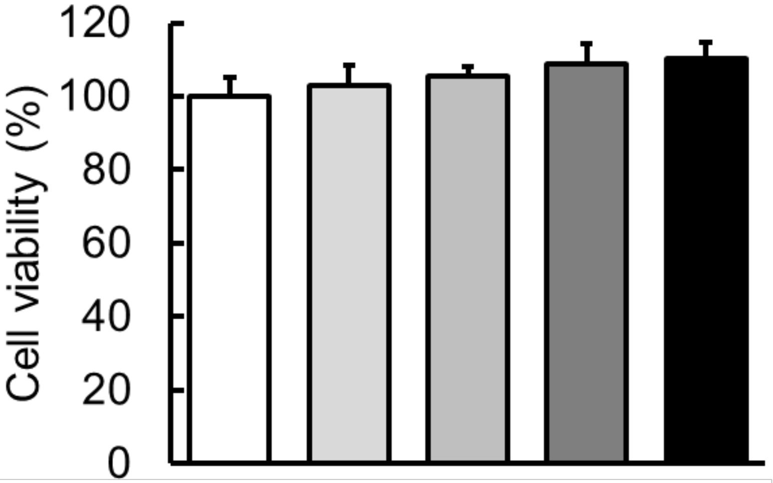

NLS-(−30)GFP (μM)	0	2.5	5.0	10	20
total lipid (mM)	0	1.2	2.4	4.8	9.7

Figure S3. Cytotoxicity of NLS-(−30)GFP-LNP treatment. Cell viability was analyzed using the WST-8 assay after treatment with NLS-(−30)GFP-LNP (equivalent to 2.5, 5.0, 10, and 20 μM NLS-(−30)GFP) for 6 h.

Table S1. Effect of the citrate buffer pH on the NLS-(−30)GFP-LNP formulation.

pH of 1 mM citrate buffer	Size (d.nm)	PdI
4.5	243 ± 42	0.272 ± 0.07
5.0	172 ± 12	0.156 ± 0.04
5.5	181 ± 12	0.198 ± 0.05
6.0	180 ± 36	0.201 ± 0.1

Lipids were composed of DOP-DEDA, DPPC, and cholesterol at a 45:10:45 molar ratio, and 1 mol% DMG-PEG5k was added. Results are presented as the mean ± SD of more than three independent experiments. A lipid concentration of 25 mM at a protein/lipid mass ratio of 1:10 was employed.

Except for the observation that the buffer at pH 4.5 yielded LNPs with a diameter of 243 nm and a PdI of 0.27, the use of citrate at other pH levels yielded no marked difference. Since the LNPs with the smallest diameter and PdI were obtained at pH 5.0, a citrate buffer at this pH was employed in the following studies.

Table S2. Effect of different volume ratios of the aqueous/organic phases on the NLS-(−30)GFP-LNP formulation.

NLS-(−30)GFP/lipid volume ratio	Size (d.nm)	PdI
3:1	1081 ± 479	0.308 ± 0.05
5:1	172 ± 12	0.156 ± 0.04
7:1	176 ± 47	0.202 ± 0.06
10:1	135 ± 5	0.183 ± 0.04

Lipids were composed of DOP-DEDA, DPPC, and cholesterol at a 45:10:45 molar ratio, and 1 mol% DMG-PEG5k was added. NLS-(−30)GFP was dissolved in 1 mM citrate buffer (pH 5.0), and lipids were dissolved in *t*-butanol. Results are presented as the mean ± SD of more than three independent experiments. A lipid concentration of 25 mM at a protein/lipid mass ratio of 1:10 was employed.

Although a 3-fold volume of the aqueous phase yielded LNPs with diameters exceeding 1000 nm, the other setups yielded diameters within 200 nm. With the expectation of an ease in LNP preparation using smaller volumes, we decided to employ a 5-fold volume of citrate buffer against t-butanol.

Table S3. Effect of the charge of the cargo protein, PEG decoration, and DOP-DEDA in the LNP formulation.

Lipids	protein	mass ratio	size (d.nm)	PdI	Corresponding LNP names and CLSM
DOP-DEDA/DPPC/Chol (45/10/45) + 1% DMG-PEG5k	NLS-(−30)GFP	1:10	172 ± 12	0.156 ± 0.04	NLS-(−30)GFP-LNPs (1:10) Figure 3
DOP-DEDA/DPPC/Chol (45/10/45) + 1% DMG-PEG5k	NLS-EGFP	1:10	140 ± 5	0.153 ± 0.2	NLS-EGFP-LNPs (1:10) Figure S3A
DOP-DEDA/DPPC/Chol (45/10/45) (no DMG-PEG5k)	NLS-(−30)GFP	1:10	2144 ± 785	0.526 ± 0.3	NLS-(-30)GFP-LNPs (1:10) (PEG(–)) Figure S3B
DOPE/DPPC/Chol (45/10/45) + 1% DMG-PEG5k	NLS-(−30)GFP	1:10	715 ± 107	0.324 ± 0.02	NLS-(-30)GFP-LNPs (1:10) (DOPE) Figure S3C

The mass ratios denote the NLS-(−30)GFP/total lipids (w/w). The formulation using the lipid composition of [DOP-DEDA/DPPC/Chol (45/10/45) + 1% DMG-PEG5k] and NLS-(−30)GFP corresponded to the NLS-(−30)GFP-LNPs (1:10) formulation (same data as in Table 1). Results are presented as the mean ± SD of more than three independent experiments.
